# Is nesting addiction medicine and hepatology care in the outpatient setting worthwhile? A retrospective case series at a single tertiary center

**DOI:** 10.1097/HC9.0000000000000555

**Published:** 2024-11-25

**Authors:** Rachit Gupta, Jecinn Wong, Christine Hallinan, Jacinta Holmes, Alexander Thompson, Adam Pastor, Yvonne Bonomo

**Affiliations:** 1 Department of Addiction Medicine, St Vincent’s Hospital, Fitzroy, Victoria, Australia; 2 Department of General Practice, Faculty of Medicine, Dentistry and Health Sciences, University of Melbourne, Parkville, Victoria, Australia; 3 Department of Medicine, Faculty of Medicine, Dentistry and Health Sciences, University of Melbourne, Parkville, Victoria, Australia

To the editor,

We read with interest the paper by Mahle et al,^1^ which prospectively examined the effectiveness of an integrated hepatology and addiction medicine service on alcohol use and hepatology outcomes in inpatients with alcohol use disorder. The authors concluded that an integrated approach improved the uptake of medical alcohol therapy, hepatic fibrosis screening, and viral hepatitis vaccination relative to a historical comparator group that received addiction medicine care alone.^1^ Although integrated care models are well established in other medical specialties, substance use disorder (SUD) and, in particular, alcohol use disorder are examples of chronic relapsing-remitting conditions that are also likely to benefit from a multidisciplinary approach.^2^^,^^3^ However, there is a paucity of literature describing such models of care in the real-world setting.

We present our retrospective review of medical records from an integrated addiction medicine and hepatology outpatient clinic at a tertiary hospital in Melbourne, Australia. This integrated clinic was held every fortnight for 4 hours where patients with SUD were reviewed concurrently by an addiction medicine specialist (or trainee) and hepatologist (or hepatology trainee). A total of 267 referrals for the clinic were received between February 2021 and September 2023, with 81 completed integrated appointments for 54 individual patients. Patient characteristics are described in Table 1. This study was approved by the St Vincent’s Hospital Melbourne Human Ethics Research Committee (reference number 044/24).

**TABLE 1:** Cohort characteristics

Total appointments, N	81
Patients, N	54
Age, mean, y	58
Sex, N (%)	
Male	35 (65)
Primary substance, N (%)	
Alcohol	54 (100)
AUD (DSM V criteria), N (%)	48 (89)
Ethnicity, N (%)	
ATSI	1 (2)
Country of birth, N (%)	
Australia	33 (61)
Polysubstance use, N (%)	16 (33)
Tobacco smoker, N (%)	24 (44)
Medical comorbidities, N (%)	41 (76)
Psychiatric comorbidities, N (%)	29 (54)
Serology	
ALT, median [IQR] (IU/L)	31 [20–51]
AST, median [IQR] (IU/L)	54 [33–87]
Total bilirubin, median [IQR] (μmol/L)	18 [12–30]
GGT, median [IQR] (IU/L)	203 [89–329]
Platelets, median [IQR] (×10^9^ /L)	167 [104–268]
MCV, median (fL)	95 [92–100]
INR, median [IQR]	1.1 [1–1.4]
TE	
TE available, N (%)	20 (37)
>12.5 kPa, N (%)	5 (20)
Median, kPa	8.7

Abbreviations: ATSI, Aboriginal and Torres Strait Islander; AUD, alcohol use disorder; INR, international normalized ratio; MCV, mean corpuscular volume; TE, transient elastography.

Five out of 54 (9%) patients were commenced on medications for alcohol use disorder (baclofen, naltrexone, or acamprosate), with a further 7/54 (13%) continued on therapy. Nine out of 54 (17%) patients were referred to specialist alcohol and drug counseling, and the majority of patients (n = 46, 85%) were given motivational interviewing during their integrated appointment. For patients who had more than 1 integrated appointment (n = 17, 31%), 8 (41%) reported alcohol cessation in the preceding month to their outpatient appointment, with a further 2 (11%) reporting a reduction in use.

Similar to Mahle et al,^1^ the integrated clinic at our hospital consisted of a highly comorbid and complex patient group reflective of a tertiary hospital patient load. This real-world model of care was a particular strength of our study. Limitations of our study included its retrospective nature, small sample size, and lack of a control group for comparison. Our model of care, along with Mahle et al, demonstrates the effectiveness of integrated models of care for patients with SUD and, in particular, alcohol use disorder.^1^ Further large-scale studies evaluating the effectiveness of integrated care models in patients with SUD/alcohol use disorder in both the inpatient and outpatient settings are needed.
